# Disparate voxel based morphometry (VBM) results between SPM and FSL softwares in ALS patients with frontotemporal dementia: which VBM results to consider?

**DOI:** 10.1186/s12883-015-0274-8

**Published:** 2015-03-13

**Authors:** Venkateswaran Rajagopalan, Erik P Pioro

**Affiliations:** Department of Electrical and Electronics Engineering, Birla Institute of Technology and Science, Pilani, 500078 Hyderabad Campus, India; Department of Biomedical Engineering, ND2, Lerner Research Institute, Cleveland Clinic, Cleveland, OH 44195 USA; Neuromuscular Center, Department of Neurology, S90, Neurological Institute, Cleveland Clinic, 9500 Euclid Avenue, Cleveland, OH 44195 USA; Department of Neurosciences, Lerner Research Institute, Cleveland Clinic, Cleveland, OH 44195 USA

**Keywords:** Grey matter atrophy, Freesurfer, FSL, SPM, VBM

## Abstract

**Background:**

Because our previous study showed disparate voxel based morphometry (VBM) results between SPM and FSL softwares in the brain of amyotrophic lateral sclerosis patients with frontotemporal dementia (ALS-FTD), we investigated which VBM results may more represent atrophy by comparing with Freesurfer’s cortical volume and thickness measures.

**Methods:**

MRI at 1.5 T was obtained during routine clinical imaging of ALS-FTD patients (*n =* 18) and in unaffected neurologic controls (*n =* 15). Gray matter (GM) VBM analysis was carried out using FSL and SPM. Cortical thickness and volume analysis was performed using Freesurfer.

**Results:**

GM volume was significantly (p < 0.05) reduced in both motor and extra motor regions in ALS- FTD when compared to unaffected neurologic controls in FSL and Freesurfer but not in SPM. Dice similarity index for cortical GM volume changes between FSL and Freesurfer was 0.30 for motor and 0.31 for non-motor regions as opposed to 0 (motor) and 0.02 (non-motor) between SPM and Freesurfer.

**Conclusion:**

GM volume changes using FSL showed similar pattern with Freesurfer cortical volume and thickness changes in contrast to SPM results. Our results suggest that, at least for our dataset, VBM results obtained using FSL software should be considered as more representative of GM atrophy.

## Background

Voxel-based morphometry (VBM) is commonly used to quantitatively assess magnetic resonance imaging (MRI) grey matter (GM) volume changes in various neurological disorders of the brain. VBM analysis allows objective and automated detection of structural changes (e.g., atrophy) in brains of patients with neurodegenerative diseases after normalizing for random shape differences [1]. It allows unbiased whole-brain analysis when the distribution of pathology is not known *a priori*. However, previous VBM studies of amyotrophic lateral sclerosis (ALS) patient brains have shown inconsistent results, including significant GM but not white matter (WM) volume changes in some reports [2,3], and the opposite in others [4,5]. Such discrepancies may be the result of a variety of factors, such as patient phenotypes, imaging parameters, software used for VBM analysis, and image contrast or statistical models employed for the analysis.

In a previous study [6] we investigated whether the inconsistent VBM results found in the ALS MRI literature were due to different softwares used for analysis, i.e. SPM or FSL. We found disparate results in VBM statistical parametric maps between SPM and FSL and that these were due to differences in segmentation, registration and statistical inference methods between the two softwares. While segmentation and registration did not play a major role in contributing to disparity between FSL and SPM results, differences in statistical methods in these softwares did. We hypothesized that the differences in statistical parametric maps between the two softwares were due to use of the threshold free cluster enhancement (TFCE) method in FSL but not in SPM. Therefore, in this study, we aimed to test this hypothesis as well as to determine whether VBM results from FSL or SPM are more representative of atrophy as compared with cortical thickness and volume measures obtained using Freesurfer software. Although not necessarily the “gold standard”, Freesurfer software-derived cortical volume and thickness measures are considered to be robust [7] for the following reasons: a) surface-based registration algorithms used in Freesurfer perform better over volume-based registration algorithms [8], b) surface models used in Freesurfer are found to be robust across different scanners and field strengths [7]. Therefore, in this study we compared statistical parametric maps of cortical thickness and volume obtained using Freesurfer with GM VBM results from FSL and SPM in ALS patients with frontotemporal dementia (ALS-FTD), and in unaffected neurologic controls.

## Methods

### Demographics

MRI data obtained at 1.5 T during routine clinical neuroimaging were approved by the Cleveland Clinic Institutional Review Board for storage and analysis as de-identified images after patients provided verbal consent. The same data set used in our previous studies [6] was analyzed in 15 unaffected neurologic controls (10 men, 5 women) aged 57.1 ± 19.2 years (mean ± SD, range 28–95 years) identified at the time of MRI, and 18 patients with ALS-FTD (5 men, 13 women) aged 66.9 ± 10 years (mean ± SD, range 52–87 years) identified by bedside (Montreal Cognitive Assessment, MoCA) or formal neuropsychometric testing. Clinical details of the ALS-FTD patients, as defined by Neary criteria [9], and neurologic controls are provided in our previous report [10].

### Image acquisition

T1-weighted data were obtained on a 1.5 T magnet (Siemens Symphony, Erlangen, Germany) using 3D magnetization-prepared rapid gradient echo (M-PRAGE) sequence. Imaging parameters were: 160 slices, voxel resolution of 1.0 × 1.0 × 1.0 mm; pulse sequence parameters were: TR = 1970 ms, TE = 4.38 ms, number of averages = 1, and scan time = 6.45 minutes.

### Data processing

VBM analysis was carried out using FSL and SPM softwares separately as described below.

### FSL approach

FSL’s standard VBM processing pipeline was adopted and the processing steps are briefly described below. An optimized VBM approach of Good *et al.* [10] was adopted with all processing steps carried out using openware FSL version 4.1.5 (http://www.fmrib.ox.ac.uk/fsl/) [9]. Data processing was divided into four major steps: 1) T1-weighted images were brain-extracted using BET [11] adopting the suggestions for using FSL’s BET outlined by Popescu *et al.* [12]. Any leftover non-brain regions of ALS-FTD patients were manually edited by painting the non-brain voxels with a mask of this created to exclude these non-brain voxels from the brain image. An experienced neurologist (EPP) with extensive neuroanatomical knowledge confirmed that only non-brain regions were removed by manual edits. Because ALS does not typically result in T1 hypointense lesions in the brain (as was the case in our ALS-FTD patients as well), no correction was applied to T1-weighted images before the segmentation step. 2) Brain extracted images were segmented into white matter, GM, and cerebrospinal fluid (CSF) volume probability maps using FAST [13]. 3) In order to avoid bias during the registration process, a study-specific GM template was created by registering into MNI152 space with the affine registration tool FLIRT [14,15]. A randomly chosen subset of subjects, as suggested in the FSL VBM user guide from both unaffected neurologic controls (*n =* 15) and ALS-FTD patients (15 subjects randomly chosen out of 18), was chosen to create the above study-specific template. After nonlinear registration using FNIRT (www.fmrib.ox.ac.uk/analysis/techrep), the resulting images were averaged to create the template. 4) All the native GM images were non-linearly reregistered to the template and modulated (affine component not included) using the Jacobian of the warp field. 5) These images were then smoothed using a full-width half-maximum (FWHM) of 7 mm, and 6) general linear model (GLM) was used to compare voxel-wise differences in GM volume between ALS-FTD and the control groups. Non-parametric statistics were performed using “randomise” with 5000 permutations and using threshold free cluster enhancement (TFCE) option either enabled or disabled (i.e. voxel-based thresholding without the TFCE option in randomise). Statistical parametric maps generated both with and without the TFCE option were then compared with SPM. Variance smoothing was not used.

### SPM approach

VBM analysis in SPM8 software was carried out using VBM8 toolbox by adopting standard VBM processing routine. The processing steps are briefly explained below: 1) estimate and write, 2) DARTEL create template, 3) DARTEL existing template, 4) normalize to MNI space, and 5) non-parametric statistics. More specifically, the first step (estimate and write) involves bias-correcting the raw T1-weighted images for inhomogeneities, extracting the brain, and segmenting it into GM, WM and CSF volume probability maps. The DARTEL create template step was used to create a customized template for our study; the same randomly chosen subjects that were used in the FSL approach were used here too. Once the study-specific template was created from the above step, the remaining subjects were registered nonlinearly to this template using DARTEL existing template module [16]. After normalizing and registering all subjects to MNI space, the resulting images were modulated (without including affine component) and smoothed using a full-width half-maximum (FWHM) of 7 mm. Finally, the smoothed images were used for statistical inference. Statistical non-parametric mapping (SnPM) with 5000 permutations without variance smoothing was used to compare voxel-wise differences in GM volumes between the ALS-FTD and control groups.

### Freesurfer approach

Cortical volume and thickness measures were estimated using the openware, Freesurfer (http://surfer.nmr.mgh.harvard.edu/). Our MR data were of high quality, and appropriate checks and edits were performed throughout the entire Freesurfer workflow (both authors evaluated/checked all the steps especially, brain segmentation, registration to Talaraich space, pial surface and GM-WM boundaries extraction results in each subject). Standard image-processing steps were adopted including: 1) correct for motion artifacts and strip skull based on a hybrid watershed/surface deformation procedure [17], 2) register images to a Talairach brain template and segment for subcortical WM and GM structures [18], 3) estimate the GM-WM boundary via a tessellation step, and subsequently perform automated topology correction, 4) optimally place GM-WM and GM-cerebrospinal fluid (GM-CSF) boundaries using surface normalization and intensity gradients, 6) after cortical models are complete, perform deformable procedures for further processing and analysis, such as surface inflation and registration to a spherical atlas [19]. Both intensity and continuity information from the entire 3D MR volume are used to produce representations of cortical thickness, where thickness is measured as the closest distance from GM-WM to GM-CSF boundary at each vertex on the tessellated surface [20].

Level of significance in Freesurfer, SPM and FSL was considered a *p* value <0.05 corrected for multiple comparisons using family-wise error rate. Covariate age was regressed out in the GLM. To quantitatively compare between SPM, FSL VBM results with Freesurfer’s cortical thickness and volume measures, we calculated percentage of voxels that reached statistical significance [6]. Similarity between statistical parametric maps of SPM, FSL with Freesurfer was obtained for motor and extra-motor regions using Dice similarity index [21-23]. In order to measure Dice similarity index, we voxelized cortical thickness and cortical volume surface statistical parametric maps to MNI space because FSL and SPM statistical parametric maps were already in MNI space. Comparing volumetric (VBM) and thickness (Freesurfer) parameters requires conversion between the two units of measure. We performed surface to volume map conversion, after Klein et al. [22], as only one resampling was necessary (i.e., Freesurfer statistical parametric maps) in comparison to volume to surface map conversion for which two resamplings would be needed (i.e., FSL and SPM statistical parametric maps). Statistical parametric maps of cortical thickness and cortical volume were sampled to the target volume (FSL and SPM statistical parametric maps were in MNI space) using mri_surf2ol, mri_aparac2aseg and mri_convert commands in Freesurfer. The resulting volume maps from Freesurfer were then used to measure Dice similarity indices with FSL and SPM statistical parametric maps. Dice similarity index measures similarity by taking the mathematical intersection (voxels common to both images in the given ROI) of similarly labeled regions (here motor cortex and non motor cortex ROI’s) between the two softwares and then dividing by the mean volumes of the two ROIs [22].

## Results

### Using TFCE in FSL

**Figure 1:**
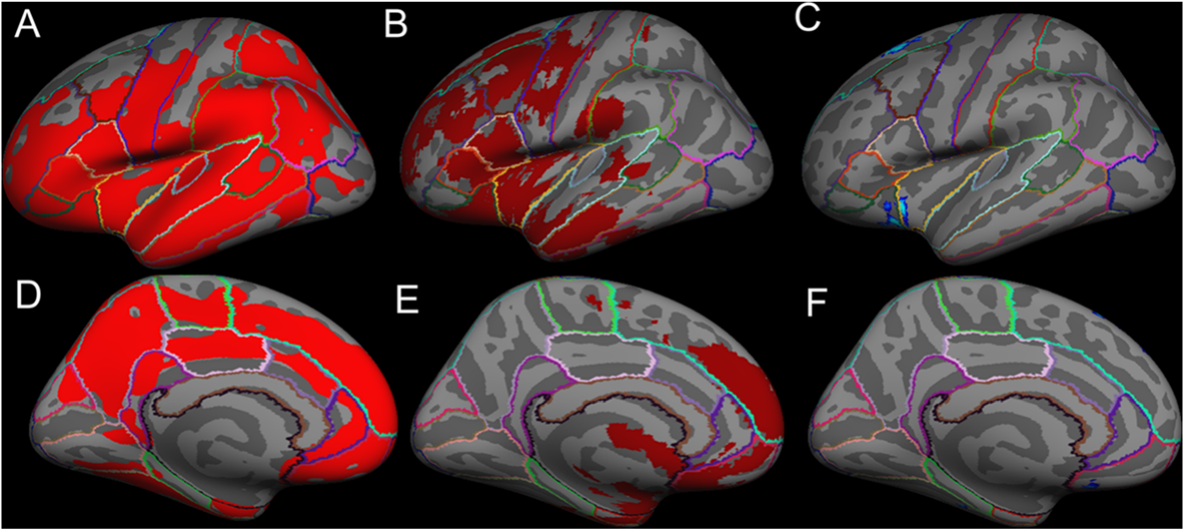
**Average inflated templates showing regions in the left hemisphere with significantly (** ***p <*** **0.05) reduced cortical volumes in ALS-FTD patients relative to unaffected neurologic controls derived with the following techniques: lateral view A) Freesurfer, B) FSL VBM, C) SPM VBM, and medial view D) Freesurfer, E) FSL VBM, F) SPM VBM.**

**Figure 2:**
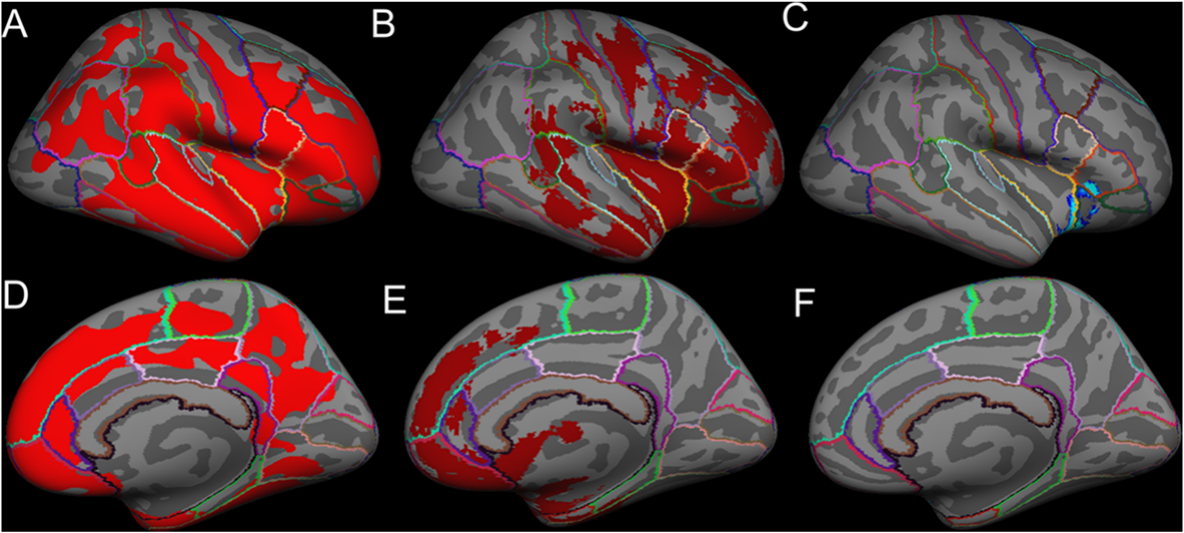
**Average inflated templates showing regions in the right hemisphere with significantly (** ***p*** **< 0.05) reduced cortical volumes in ALS-FTD patients relative to unaffected neurologic controls derived with the following techniques projected in lateral view A) Freesurfer, B) FSL VBM, C) SPM VBM, and medial view D) Freesurfer, E) FSL VBM, f) SPM VBM.**

**Figure 3:**
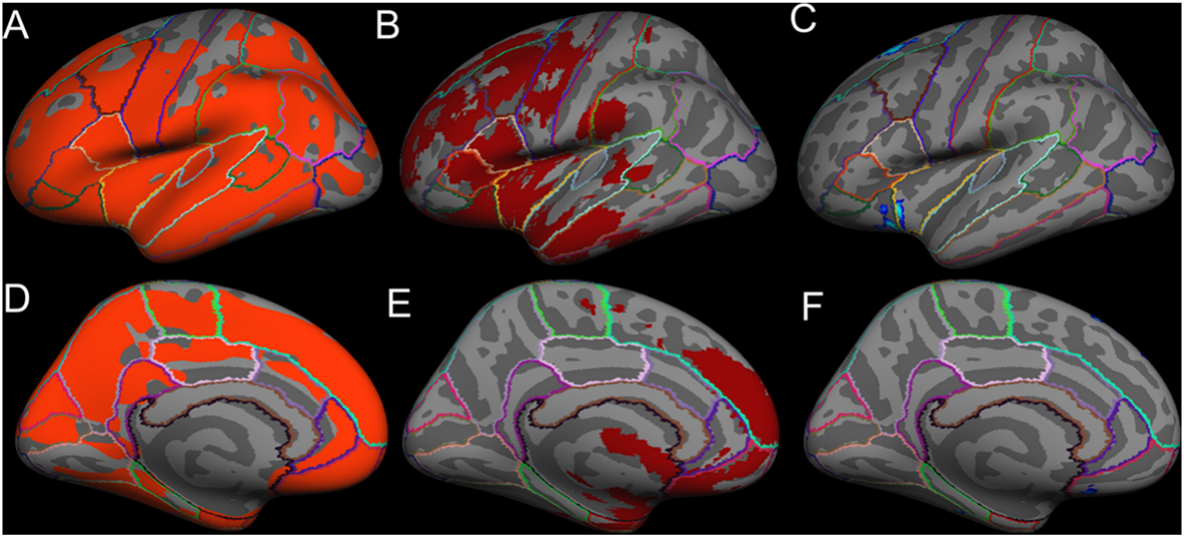
**Average inflated templates showing regions in the left hemisphere with significant (** ***p*** **< 0.05) cortical thinning in ALS-FTD patients relative to unaffected neurologic controls derived with the following techniques projected in lateral view A) Freesurfer, B) FSL VBM, C) SPM VBM, and medial view D) Freesurfer, E) FSL VBM, and F) SPM VBM.**

**Figure 4:**
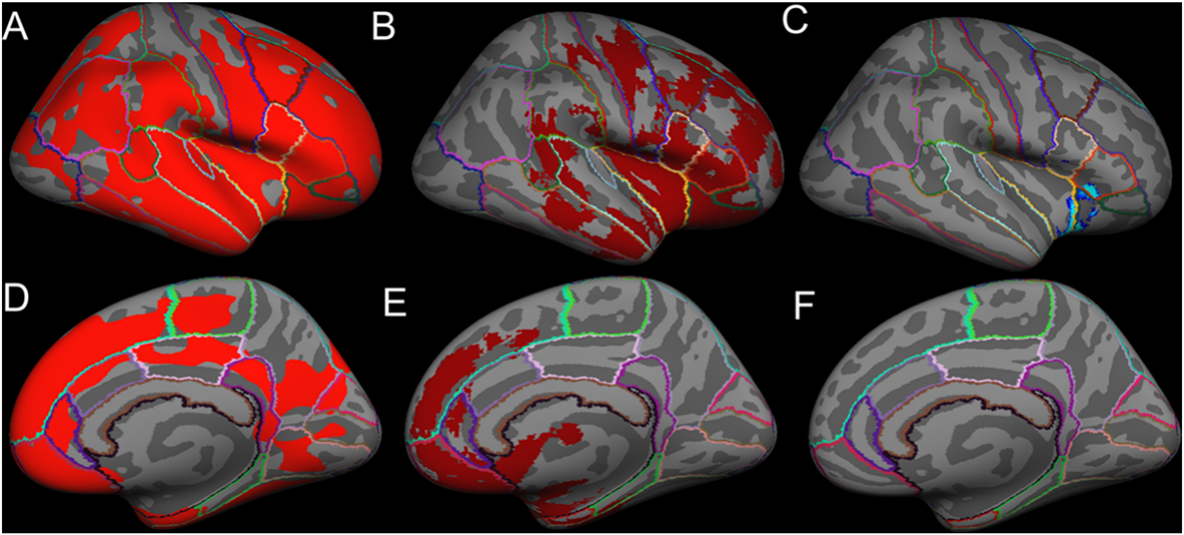
**Average inflated templates showing regions in the right hemisphere with significant (** ***p*** **< 0.05) cortical thinning in ALS-FTD patients relative to unaffected neurologic controls derived with the following techniques projected in lateral view A) Freesurfer, B) FSL VBM, C) SPM VBM, and medial view D) Freesurfer, E) FSL VBM, F) SPM VBM.**

Using FSL with TFCE “on” and Freesurfer, cortical *GM volume* was significantly reduced in both motor and extra-motor regions, whereas SPM8 showed significant GM changes in only extra-motor regions of ALS-FTD patients, when compared to unaffected neurologic controls (*p <* 0.05), as shown in Figures 1 and 2. In addition, significant reduction in *cortical thickness* was also observed in motor and extra motor regions (almost same regions where cortical volume changes were observed) of the ALS-FTD group compared to unaffected neurologic controls, as shown in Figure 3 and 4.

The cortical regions found to be atrophied in both FSL VBM and in Freesurfer’s cortical volume, cortical thickness measures included the primary motor cortex (precentral gyrus) and the following extra-motor gyri: superior frontal, rostral middle frontal, caudal middle frontal, postcentral, pars opercularis, pars triangularis, pars orbitalis, lateral orbitofrontal, insula, superior temporal, middle temporal, supramarginal, inferior temporal, inferior parietal, lateral occipital, superior parietal, lateral occipital, cuneus, lingual, pericalcrine, precuneus, paracentral, superior frontal, medial orbitofrontal, rostral anterior cingulate, posterior cingulate, and isthmus cingulate. On the other hand, SPM VBM showed significant atrophy only in the insula, superior frontal, medial orbitofrontal and lateral orbital gyri. The extent of atrophy seen in each of the aforementioned regions differed slightly between FSL VBM and Freesurfer’s measures.

Dice similarity index was used to assess the similarity between SPM or FSL and Freesurfer in motor and extra-motor regions, where a higher number indicates greater similarity; we combined all aforementioned extra-motor regions into a single “extra-motor region” to study similarity measures. Similarity indices for cortical GM *volume* changes (in right and left hemispheres) between FSL and Freesurfer were 0.3 for motor cortex and 0.315 for extra-motor cortex, while between SPM and Freesurfer were 0.0 for motor cortex and 0.02 for extra-motor cortex (given in Table 1). No significant changes in cortical *area* measures were found in any of the brain regions between ALS-FTD patients and unaffected neurologic controls.

**Table 1:** **Dice similarity index values of statistical parametric maps between the softwares**

**Dice similarity index**	**FSL (TFCE ON) vs Freesurfer**	**FSL (TFCE OFF) vs SPM**	**FSL (TFCE OFF) vs Freesurfer**	**SPM vs Freesurfer**
	0.3 (motor cortex), 0.32 (extra-motor cortex)	0.04 (left and right hemispheres)	0.0 for motor cortex and 0.0 for extra-motor cortex	0.0 (motor cortex), 0.02 (extra-motor cortex)

Using FSL VBM approach, nearly 22.5% of brain voxels reached statistical significance (i.e. atrophy) when compared to 0.81% in SPM VBM approach. In order to measure the percentage of voxels that reached statistical significance in cortical volume and thickness measures from Freesurfer, the surface significance maps were converted to volume images. Percentages of total voxels significantly reduced (in right and left hemispheres) with Freesurfer were 64.5% by cortical volume and 72.3% by cortical thickness measures.

### Not using TFCE in FSL

As in SPM, FSL without TFCE “on” showed significant GM changes only in extra-motor regions of ALS-FTD patients compared to unaffected neurologic controls (*p <* 0.05). However, the number of cortical regions and the extent to which they were atrophied were even lower in FSL's statistical parametric map when not using TFCE. Only 0.03% of brain voxels reached statistical significance when compared to 0.81% in SPM VBM approach.

Dice similarity index was used to assess the similarity between SPM or FSL with Freesurfer in motor and extra-motor regions of right and left hemispheres; as previously, we combined all aforementioned extra-motor regions into a single “extra-motor region” to study similarity measures. The similarity indices for cortical GM *volume* changes between FSL and Freesurfer were 0.0 for motor cortex and 0.0 for extra-motor cortex, while between SPM and Freesurfer were 0.0 for motor cortex and 0.02 for extra-motor cortex. The similarity index was only 0.04 (for left and right hemispheres, we did not calculate for motor and extra-motor cortex here because no significant atrophy was observed in motor cortex in SPM results) between SPM and FSL (given in Table 1).

## Discussion

VBM results in the ALS literature have been conflicting and inconclusive. In our previous study [6], we compared VBM results in ALS-FTD patients derived from both SPM and FSL approaches to determine if this would explain the differences. We found disparate statistical parametric maps between FSL and SPM VBM approaches, which appear to arise from differences in registration and statistical approaches between the softwares. In that previous study, however, we did not determine whether use of the TFCE method in FSL statistics was responsible for the disparate results, and also which these two software approaches resulted in the more representative VBM results. In order to address these questions, we compared SPM and FSL VBM results with Freesurfer’s cortical volume and thickness measures. Although arguably not the “gold standard” measure, Freesurfer appears to take a more robust approach than volume-based measures [22]. We used Freesurfer to compare with SPM and FSL VBM results because it: a) uses surface geometry to perform intersubject registration for group comparisons giving better matching of homologous cortical regions; b) is a commonly used openware to study cortical atrophy in various neurological disorders of the brain, including a report of patients with ALS in which cortical thinning was a better indicator of neurodegeneration than cortical volume loss [24].

The two main findings of this study included: a) a large disparity in results between SPM and FSL when the TFCE option was enabled in FSL compared to when it was disabled. Specifically, when TFCE was used, the Dice similarity index between FSL and Freesurfer was 0.3 compared to being significantly reduced to almost 0 when it was not used; b) disparity in statistical parametric maps of SPM and FSL whether TFCE was enabled or not (the Dice similarity index remained very low).

Differences in image processing algorithms and statistical methods used in these aforementioned softwares could underlie the disparate atrophy findings. Klein et al. [8] evaluated performance of 14 different non-linear registration algorithms used in several software packages and found that SPM8’s DARTEL registration algorithm (used in this study) gave good results when compared to FSL’s FNIRT non-linear registration algorithm. We also found that differences in registration algorithms and statistical approaches between FSL and SPM led to large disparity in VBM results [6]. Similarly, the disparities we have found between Freesurfer and SPM or FSL results can be attributed to differences in their registration algorithms. The registration method used in Freesurfer is surface-based (vertex-based) rather than volume-based (voxel-based) as in FSL and SPM, and differences arising from these two registration methods have been previously demonstrated [22]. Other possible reasons for the differences in our results include: a) inclusion of both cortical thickness and cortical foldings (gyri and sulci) in VBM measures such that adjacent gyri could be mistaken for a single region and thereby cause erroneously high GM values; b) differences despite employing a non-parametric permutation based statistical approach in all softwares used in this study. Even when TFCE was disabled no similarity was found between the softwares (Dice similarity index given in Table 1). FSL randomise uses threshold-free cluster enhancement (TFCE) whereas, SnPM does not. In Freesurfer, cluster wise threshold option was used. Because of the relatively close similarity in the statistical parametric maps of FSL (when TFCE is used) and Freesurfer, and that TFCE is a hybrid method with the benefits of cluster-based maps without the need to specify the suprathreshold, we prefer the FSL over the SPM approach for VBM analysis of our dataset.

Although an extensive comparison of each of the image processing steps across these softwares would provide more detailed information on such intrinsic differences, it is beyond the scope of this study (because the processing steps include hundreds of potentially adjustable parameters). We used the standard default parameter settings in SPM, FSL and Freesurfer processing steps, as provided by these software packages, in order to stay within the scope of the study. A more detailed analysis could be carried out in the future to determine how altering the multiple parameter settings of the image pre-processing steps would affect VBM and Freesurfer results. In addition, there are several cortical thickness analysis pipelines openly available, some of which are volume-based rather than surface-based. Occurrence of disparate results between VBM approaches used suggests similar differences may also occur between the various cortical thickness approaches. Therefore, use of cortical thickness analytic methods other than Freesurfer for detailed comparisons may be appropriate.

## Conclusion

We compared GM VBM changes between ALS-FTD patients and unaffected neurologic controls using two popular image analysis software programs (SPM and FSL), with Freesurfer’s cortical thickness and volume measures. Our results demonstrate that the pattern of GM volume changes identified in different brain regions using FSL VBM and Freesurfer is almost identical when TFCE is used in FSL. Because TFCE has the advantage of being a hybrid method without the need to specify a suprathreshold, we regard FSL’s GM VBM results using TFCE as more correct, at least for our dataset and imaging parameters. More studies are needed to determine which algorithms are the most appropriate for processing these types of imaging data.

## Abbreviations

ALS: Amyotrophic lateral sclerosis
ALS–FTD: Amyotrophic lateral sclerosis with frontotemporal dementia
BET: Brain extraction tool
DARTEL: Diffeomorphic anatomical registration through exponentiated lie algebra
FAST: FMRIB’s automated segmentation tool
FLIRT: FMRIB’s linear image registration tool
FMRIB: The Oxford Centre for Functional MRI of the brain
FNIRT: FMRIB’s nonlinear image registration tool
FSL: FMRIB software library
FWER: Family wise error rate
FWHM: Full-width half-maximum
GLM: General linear model
GM: Grey matter
MoCA: Montreal cognitive assessment
MPRAGE: Magnetization prepared rapid gradient echo
MRI: Magnetic resonance imaging
SnPM: Statistical non-parametric mapping
SPM: Statistical parametric mapping
TFCE: Threshold-free cluster enhancement
VBM: Voxel based morphometry
